# Folk taxonomy and indigenous names for frogs in Zululand, South Africa

**DOI:** 10.1186/s13002-019-0294-3

**Published:** 2019-03-26

**Authors:** Fortunate M. Phaka, Edward C. Netherlands, Donnavan J. D. Kruger, Louis H. Du Preez

**Affiliations:** 1 African Amphibian Conservation Research Group, Unit for Environmental Sciences and Management, North-West University, Private Bag X6001, Potchefstroom, 2520 South Africa; 2 Laboratory of Aquatic Ecology, Evolution and Conservation, University of Leuven, Charles Debériotstraat 32, Leuven, B-3000 Belgium; 3 Focus area: Self Directed Learning, Faculty of Education, North-West University, Private Bag X6001, Potchefstroom, 2520 South Africa; 4 South African Institute for Aquatic Biodiversity, Somerset Street, Grahamstown, 6140 South Africa

**Keywords:** Anura, Ethnotaxonomy, Indigenous knowledge, Maputaland-Pondoland-Albany, Taxonomy

## Abstract

**Background:**

We use taxonomy to organize the world into recognizable units. Folk taxonomy deals with the naming and classification of organisms through culture. Unlike its scientific counterpart, folk taxonomy is mostly undocumented, the Zoological Code of Nomenclature does not regulate it, and the resulting names are specific to each culture. A growing body of literature is steadily shedding light on the principles underlying this pre-scientific taxonomy. Vernacular names can be an instrument to increase participation of non-scientists in biodiversity matters. In South Africa, great strides have been made in standardizing and increasing relatability of vernacular amphibian names in English and Afrikaans. However, there is a need to achieve the same with the country’s autochthonous languages which are used by a majority of the population.

**Methods:**

This study investigates amphibian-related folk taxonomy using a semi-structured interview process in KwaZulu-Natal’s Zululand region and  pilots methods of applying folk taxonomy principles to compile a comprehensive list of standardized indigenous frog names.

**Results:**

Folk taxonomy in Zululand is systematic, developed, and bears similarities to other indigenous taxonomies around the world. Similarities also exist between folk and scientific taxonomy. Six uninomial indigenous names were found to be used for the 58 amphibian species occurring in the study area. The 58 species were assigned individual indigenous names using folk taxonomy guidelines supplemented with guidelines for modern taxonomies.

**Conclusions:**

There is a gap in the documentation and investigation of amphibian folk taxonomy in South Africa. Standardization of indigenous frog names is required to increase their universality. Similarities between folk and modern taxonomies allow for supplementation of indigenous guidelines when compiling a comprehensive indigenous species list. Through this study, social inclusion in wildlife matters is increased, indigenous knowledge systems are promoted, and a contribution is made to the development of an indigenous South African language.

**Electronic supplementary material:**

The online version of this article (10.1186/s13002-019-0294-3) contains supplementary material, which is available to authorized users.

## Background

Taxonomy is the manifestation of a human need to organize the world into recognizable units [14, 22]. Humans recognize biodiversity and classify related living organisms in similar ways [9]. Nomenclature is motivated by communication, and to share knowledge about organisms we need to be able to identify them in ways that give meaning to a conversation. For this reason, it is essential that unique names are assigned to each organism. Unique species names are also vital to biodiversity conservation [27]. Naming ambiguities could lead to costly conservation interventions being wasted on non-threatened species that share names with species facing extinction.

Individual species names can be assigned using scientific taxonomy, and the International Code of Zoological Nomenclature (ICZN Code) seeks to ensure the standardization and universality of resulting names [20]. Folk taxonomy is a pre-scientific type of naming and classification system rooted in culture [7]. Ethnotaxonomy is a field of study dedicated to understanding the principles underlying folk taxonomy [13]. Folk taxonomic names have localized use due to culture’s specificity. One folk name is often used in reference to several species [5, 17, 38]. Furthermore, folk nomenclature is based on onomatopoeia, description, imagery [10, 24], and phonaesthesia; the non-arbitrary sound-meaning associations of movement, size, and shape [10]. Some studies have explored whether folk taxonomy uses a utilitarianist (motivated by utilitarian value) or an intellectualist (cognitively motivated) approach to classification and nomenclature [8].

Despite being pre-scientific, folk taxonomy is systematic and developed [7, 32]. Researchers have demonstrated that folk names are not just abstract notions, but condensed forms of knowledge with multiple applications [19]. Folk taxonomies contain a richness of information on the biology, ecology, and ethology of several faunal and floral taxa [26]. Ulicsni et al. [38] reported that scientific names for some Hungarian invertebrate species originate from folk names. Folk taxonomy’s fundamental organizing principles provided Linnaeus with a basis for formalizing the hierarchical structure of scientific taxonomy [32]. Unlike Linnaean taxonomy, folk taxonomy is mostly undocumented and at risk of being lost unless it is preserved. The urbanization of traditional societies is leading to a decrease in folk taxonomy usage [18]. The diminishing vocabulary of many languages makes it necessary to preserve and update vernacular species names for future use [25].

Collecting these names requires recording the speaker’s home language and the spoken dialect, as well as the location from where information was obtained [41]. Early collection and investigation of vernacular names for South African amphibians revealed several issues affecting the use of English common names. These include multiple authors using multiple names for one species, one author using multiple names for the same species in different publications, and names that are inappropriate for the named species [39]. These naming issues create confusion, especially for non-scientists, and thus, standardization of vernacular names for frogs is required [21, 40].

Jacobsen [21] and Van Dijk [40] suggested the following guidelines for increasing universality of English and Afrikaans names for frogs. (1) The vernacular name should preferably relate to its scientific counterpart. (2) References to calls, habitats, and localities should be avoided unless species are restricted to localities or have distinctive traits. (3) A person’s name should only be used when necessary. Based on the findings from an investigation of the vernacular naming problems for South African frogs, Passmore and Caruthers [28, 29] published the most appropriate English names and began a process of standardizing common names for frogs. Those published names were based on the following guidelines (see [28]). (1) Give priority to previously published names and only replace them if they are inappropriate. (2) Select the most appropriate if more than one name has been published. (3) Calls, habitats, localities, and essential aspects of morphology should preferably be used whenever there is a need to coin new names. (4) No common names should be allocated to subspecies. English names from Passmore and Caruthers [28, 29] were revised and published by Minter et al. [23] along with their Afrikaans equivalents and six indigenous names (one xiTsonga, three sePedi, and two seSotho names). Du Preez and Carruthers [15, 16] updated the Afrikaans and English species list from Minter et al. [23] with names of several newly described species. Tarrant [36] increased the number of published indigenous names by publishing isiXhosa and isiZulu names for 55 South African frog species.

The strides made in standardizing English and Afrikaans frog names created a gap to achieve the same for the other South African languages spoken by a majority of the country’s population. This study aimed to investigate amphibian folk taxonomy and supplement its guidelines with their modern knowledge counterparts to compile a comprehensive list of isiZulu names for Zululand’s frogs. The process enables previously undocumented names to be published, thus initiating their preservation and standardization.

The need to bridge this vernacular name gap is further prompted by South Africa's National Biodiversity Strategy and Action Plan (NBSAP) which states that biodiversity provides South Africans with a rich heritage of nature-based cultural traditions and further reiterates the significance of wildlife to the country’s cultures [34]. Having this culturally significant biodiversity mostly documented and investigated in only two of the country’s 11 official languages excludes a significant portion of the population from participation in biodiversity matters. The standardization of indigenous names fosters inclusion of previously marginalized languages in amphibian conservation and increases usefulness and stability of the names as has been done with scientific, English, and Afrikaans names. Researching amphibian indigenous taxonomy in itself increases participation of local community members in amphibian diversity matters and results of this research will help decrease future naming ambiguities when involving locals in amphibian diversity matters.

## Materials and methods

The current study of amphibian-related folk taxonomy was carried out in South Africa’s Zululand region. This region in the North-eastern part of KwaZulu-Natal (KZN) province falls within the Maputaland-Pondoland-Albany biodiversity hotspot. Data was collected using a semi-structured questionnaire (Additional file 1) simultaneously administered to a group of participants aged 18 to 55 during an amphibian diversity workshop. An opportunity sample was obtained by asking members of the Zululand community with interest in wildlife matters to volunteer their participation in the workshop and study. The first group of 10 community members participated on 28 November, and the second group of 3 participated on 1 December 2016. This amphibian diversity workshop, held at Tembe Elephant Park, constituted the social component for an amphibian diversity study. Ethical clearance for this amphibian diversity study was obtained from the North-West University Institutional Research Ethics Regulatory Committee (ethics number NWU-00348-16-A5). The sample of 13 participants (3 female and 10 male) are native isiZulu speakers of the same dialect who reside in 5 Zululand regions with similar environmental conditions. Their socioeconomic status varied; five were permanently employed and three were temporarily employed by nature reserves around the Zululand area, three were unemployed, and the remaining two were students. Additional data was obtained from a multilingual amphibian handbook by Tarrant [36].

Participants were shown reference photographs of all frog species that occur in the study area and collectively asked once whether isiZulu names for those species were available. With each name obtained, the group of participants was again asked once to provide the reasoning or meaning behind the name so as to better understand the taxonomic principles used. This second question prompted discussions among the group. When the participants did not agree on a particular name, they would deliberate on the nomenclature until they arrived at a conclusion everyone approved. In addition to enabling collection of data on the taxonomic principles in use, these discussions presented an opportunity to collect data on the local knowledge of amphibians beyond their taxonomy.

Following the investigation of the Zululand community’s amphibian folk taxonomy, and in the absence of published indigenous naming guidelines, findings from this study were supplemented with English and Afrikaans species’ name guidelines. Using a combination of the studied folk taxonomy guidelines and the supplementary guidelines from Vesey-FitzGerald [41], Jacobsen [21], Passmore and Carruthers [28], and Van Dijk [40] all 58 species within the study area were assigned individual isiZulu names. Formulation of individual names involved expansion of indigenous names obtained from the Zululand community and modification of names published by Tarrant [36] to increase their appropriateness. This assignment of individual species names was carried out after the amphibian diversity workshop with the assistance of Mr. Bongani Mkhize, a Ndumo Game Reserve field guide who was among the 13 participants surveyed for this study. The rigor of the ICZN Code [20] was applied to the collected frog names and formulated individual species names to determine overlaps in folk and scientific taxonomy. At the end of the above process, the indigenous names were published next to their scientific and English counterparts in a popular publication (see [30]), thus adding to the tally of published isiZulu names and modifying existing names to increase their appropriateness.

## Results

This anuran folk taxonomy investigation in Zululand found the following guidelines to be in use. (1) Classification and nomenclature are based on habit, habitat, or appearance. (2) Classification is limited to genus or higher taxa, and no individual species names are assigned. (3) Advertisement calls are unreliable for naming purposes as frogs are mostly heard and seldom seen calling. When the above indigenous taxonomy guidelines are supplemented with their modern knowledge counterparts (see [21, 28, 40, 41]), the resulting guidelines are as follows: (1) avoid coining completely new names and give priority to existing appropriate names. (2) Formulating individual species names should rather involve modification or extension of existing indigenous names to improve their meaning. (3) Habit, habitat, or appearance should preferably be used whenever there is a need to coin a new name. (4) Use of call descriptions in names should be limited to frogs that are commonly observed calling. (5) Wherever possible, the coined indigenous names should bear a similar meaning to scientific names or other vernacular names published in a different language. (6) Dialects of the language in use should be considered and species’ names made understandable across different dialects of the same language.

### Indigenous uninomial frog names

**Fig. 1:**
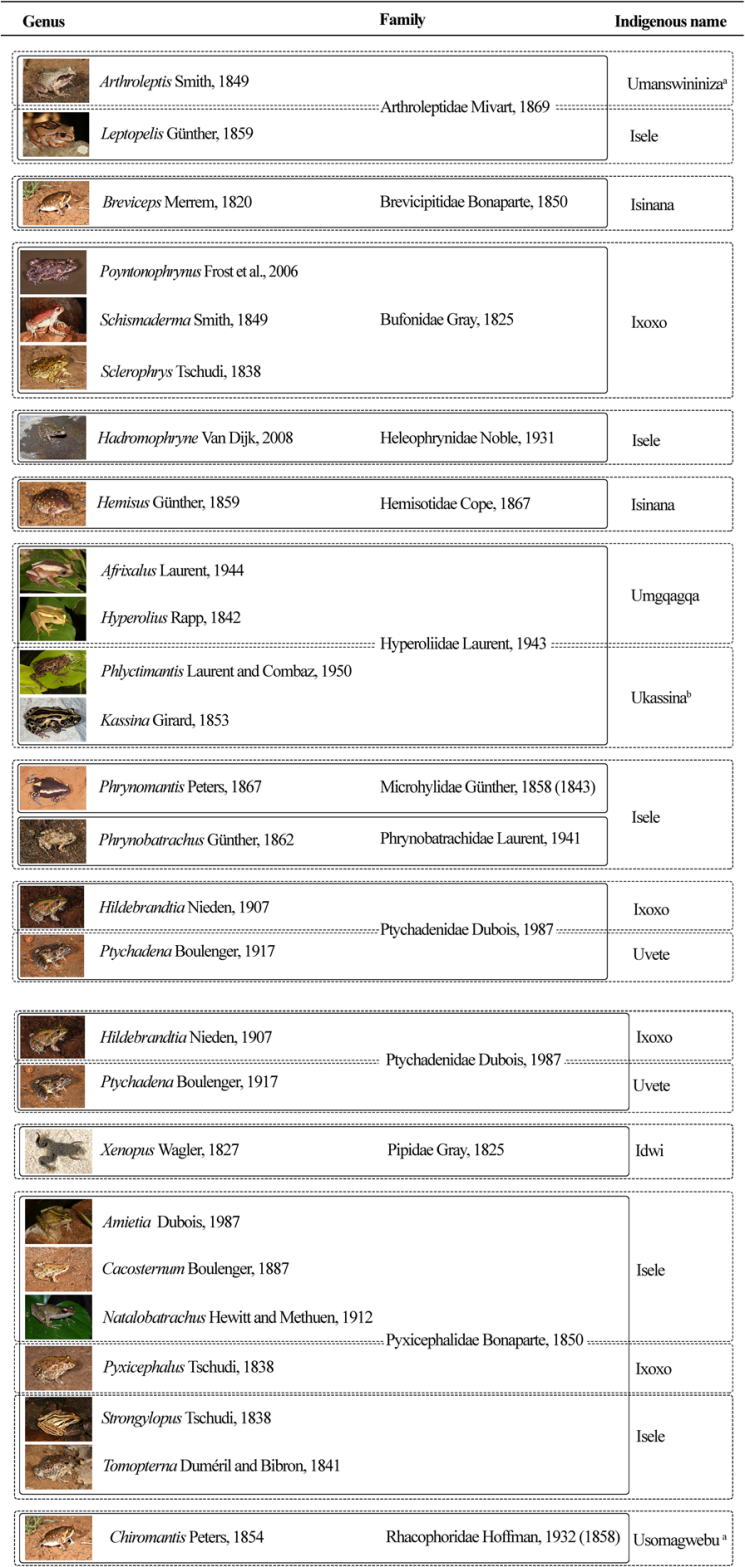
Correspondence of amphibian scientific taxa (24 genera, 12 families) with their folk taxonomy equivalents. Six folk names were obtained through a survey of 13 Zululand community members, 3 folk names were obtained from Tarrant [36], and 1 folk name was borrowed from an existing frog name. Superscript letter “a” denotes the name modified from Tarrant [36] with the assistance of Mr. Bongani Mkhize. Superscript letter “b” denotes name borrowed from existing English generic and common name. *Poyntonophrynus* sp. photograph was used with permission from LS Minter

The survey of 13 participants revealed that 6 isiZulu uninomial names are used for amphibian species in the study area (Fig. 1). The uninomial umgqagqa is generally used for reed frogs and leaf-folding frogs (e.g., Hyperoliidae). Isinana refers to fossorial frogs (e.g., Breviceptidae), while idwi is used as the uninomial name for aquatic frogs (Pipidae). Grass frogs (Ptychadenidae) are referred to as uvete. Frogs with granular or warty skin (e.g., Bufonidae) are generally called ixoxo. Iselesele, which is sometimes shortened to isele, generally refers to the smoother-skinned species (e.g., Microhylidae) and all species not included in the other five uninomial names. Ixoxo and iselesele are used interchangeably as general terms for all anurans, similar to the English term “frogs”. No names were assigned to individual frog species in Zululand.

An additional three uninomial names, umanswininiza which refers to squeakers (*Arthroleptis* spp.), usomagwebu used in reference to foam-nest frogs (*Chiromantis* sp.), and ukassina which refers to frogs of the genera *Kassina* and *Phlyctimantis*, were included in the tally of isiZulu frog uninomial names. Umanswininiza and usomagwebu are obtained from isiZulu species names published by Tarrant [36]. Ukassina is borrowed from the generic and common name *Kassina* and was modified with the assistance of Mr. Bongani Mkhize. The word umanswininiza translates to squeaker and is an onomatopoeic reference to the genus’ advertisement calls, while usomagwebu means the maker or producer of foam.

### Uninomial names: overlaps between folk and scientific taxonomy

Folk and scientific taxonomy in Zululand have a similar intellectualist approach, as classification and nomenclature of frog species in the study area are not based on utilitarian value. Application of the ICZN Code [20] to the nine isiZulu uninomial names reveals further overlaps between folk and scientific taxonomy. Similar results are obtained even when the three additional uninomial names are excluded from analysis. The indigenous names at the least represent folk-generic taxa; single words that group frog species according to their habits, habitats, or appearance. This is in line with the ICZN Code’s Article 4, which provides that scientific names of taxon ranked higher than the species group should be uninomial.

The six collected uninomial names and the three additional names are indigenous equivalents of scientific taxonomy’s genus or family levels (Fig. 1). Idwi corresponds perfectly with the taxa *Xenopus* and Pipidae. Uvete is the folk taxonomic match of the *Ptychadena* genus. When the additional uninomial names are taken into consideration, then three of the nine folk taxa correspond perfectly with scientific taxonomic ranks (Fig. 1). The remainder of the folk taxa corresponds with multiple scientific genera and/or families.

### Assigning isiZulu names to individual frog species

All 58 species of amphibians occurring in the Zululand region have been assigned individual isiZulu names (Table 1) which were published next to their scientific and English counterparts in Phaka et al. [30]. These indigenous species names bear a similar meaning to scientific names and/or vernacular (English or Afrikaans) names in recently published works (see [15, 16, 23]). Isizulu names for 30 Zululand frog species were formulated by extending the documented uninomial names. Names for the remaining 28 species were modified from species names published in Tarrant [36].

**Table 1:** IsiZulu names assigned to 58 Zululand amphibian species published in Phaka et al. [30]

Isizulu name (scientific name)	
1. Umanswininiza onyawo zingamafosholo (*Arthroleptis stenodactylus* Pfeffer, 1893)	
2. Umanswininiza wasehlathini^a^ (*Arthroleptis wahlbergii* Smith, 1849)	
3. Isele lasezihlahleni elinsundu^a^ (*Leptopelis mossambicus* Poynton, 1985)	
4. Isele lasezihlahleni laseNatali (*Leptopelis natalensis* (Smith, 1849))	
5. Isinana sasehlathini^a^ (*Breviceps adspersus* Peters, 1882)	
6. Isinana sikaBilbo^a^ (*Breviceps bagginsi* Minter, 2003)	
7. Isinana sakwaPhinda (Breviceps carruthersi Du Preez, Netherlands, and Minter, 2017)	
8. Isinana saseMozambique (*Breviceps mossambicus* Peters, 1854)	
9. Isinana sakwaNdumo (Breviceps passmorei Minter, Netherlands, and Du Preez, 2017)	
10. Isinana sekhwela/somtshingo (*Breviceps sopranus* Minter, 2003)	
11. Ixoxo elifishane (*Poyntonophrynus fenoulheti* (Hewitt and Methuen, 1912))	
12. Ixoxo elibomvu^a^ (*Schismaderma carens* (Smith, 1848))	
13. Ixoxo eliklabalasayo^a^ (Sclerophrys capensis Tschudi, 1838)	
14. Ixoxo eliluhlaza okotshani (Sclerophrys garmani (Meek, 1897))	
15. Ixoxo lembodlomane^a^ (Sclerophrys gutturalis (Power, 1927))	
16. Ixoxo lomhlane oyisicaba (Sclerophrys pusilla (Mertens, 1937))	
17. Isele lasempophomeni (*Hadromophryne natalensis* (Hewitt, 1913))	
18. Isinana esimabhadubhadu^a^ (*Hemisus guttatus* (Rapp, 1842))	
19. Isinana esipendiwe (*Hemisus marmoratus* (Peters, 1854))	
20. Umgqagqa oyigolide (*Afrixalus aureus* Pickersgill, 1984)	
21. Umgqagqa othambile (*Afrixalus delicatus* Pickersgill, 1984)	
22. Umgqagqa omkhulu^a^ (*Afrixalus fornasini* (Bianconi, 1849))	
23. Umgqagqa i-Argus^a^ (*Hyperolius argus* Peters, 1854)	
24. Umgqagqa opendiwe^a^ (*Hyperolius marmoratus* Rapp, 1842)	
25. Umgqagqa ka-Pickersgill (*Hyperolius pickersgilli* Raw, 1982)	
26. Umgqagqa omude (*Hyperolius poweri* Loveridge, 1938)	
27. Umgqagqa weminduze^a^ (*Hyperolius pusillus* (Cope, 1862))	
28. Umgqagqa wemigqa ephuzi (*Hyperolius semidiscus* Hewitt, 1927)	
29. Umgqagqa oluhlaza okotshani^a^ (*Hyperolius tuberilinguis* Smith, 1849)	
30. Ukassina wemilenze ebomvu (Phlyctimantis maculatus (Duméril, 1853))	
31. Ukassina obhadlayo^a^ (*Kassina senegalensis* (Duméril and Bibron, 1841))	
32. Isele elisanjoloba elinemigqa^a^ (*Phrynomantis bifasciatus* (Smith, 1847))	
33. Isele lechibi lasempumalanga Afrika (*Phrynobatrachus acridoides* (Cope, 1867))	
34. Isele lechibi elifishane^a^ (*Phrynobatrachus mababiensis* FitzSimons, 1932)	
35. Isele lechibi elihonayo^a^ (*Phrynobatrachus natalensis* (Smith, 1849))	
36. Ixoxo elihlotshisiwe^a^ (*Hildebrandtia ornata* (Peters, 1878))	
37. Uvete olujwayelekile (*Ptychadena anchietae* (Bocage, 1868))	
38. Uvete olunomugqa obanzi (*Ptychadena mossambica* (Peters, 1854))	
39. Uvete lwaseNile^b^ (Ptychadena nilotica (Seetzen, 1855))	
40. Uvete olunempumulo ecijile^a^ (*Ptychadena oxyrhynchus* (Smith, 1849))	
41. Uvete olunemigqa^a^ (*Ptychadena porosissima* (Steindachner, 1867))	
42. Uvete olufishane (*Ptychadena taenioscelis* Laurent, 1954)	
43. Idwi elijwayelekile^a^ (*Xenopus laevis* (Daudin, 1802))	
44. Idwi lika-M*ü*ller (*Xenopus muelleri* (Peters, 1844))	
45. Isele elithambile elijwayelekile (*Cacosternum boettgeri* (Boulenger, 1882))	
46. Isele elithambile laKwaZulu (Cacosternum nanogularum Channing et al. 2013)	
47. Isele elithambile elisathusi^a^ (*Cacosternum nanum* Boulenger, 1887)	
48. Isele elithambile elinemigqa (*Cacosternum striatum* FitzSimons, 1947)	
49. Isele lase-Kloof (*Natalobatrachus bonebergi* Hewitt and Methuen, 1912)	
50. Isele lasemfuleni elijwayelekile^a^ (Amietia delalandii (Duméril and Bibron, 1841))	
51. Inkunzi yexoxo (*Pyxicephalus edulis* Peters, 1854)	
52. Isele lasemfuleni elinemidwa^a^ (*Strongylopus fasciatus* (Smith, 1849))	
53. Isele lasemfuleni eligqafazayo^a^ (*Strongylopus grayii* (Smith, 1849))	
54. Isele lasesihlabathini elinemigqa (*Tomopterna cryptotis* (Boulenger, 1907))	
55. Isele lasesihlabathini elingqongqozayo^a^ (*Tomopterna krugerensis* Passmore and Carruthers, 1975)	
56. Isele lasesihlabathini laseNatali^a^ (*Tomopterna natalensis* (Smith, 1849))	
57. Isele lasesihlabathini likaTandy (*Tomopterna tandyi* Channing and Bogart, 1996)	
58. Usomagwebu waseningizimu^a^ (*Chiromantis xerampelina* Peters, 1854)	

A total of 30 new isiZulu species names were newly formulated, 28 were modified from published names, and 6 folk generic names were obtained through interviewing 13 Zululand community members

^a^Name modified from Tarrant [36] with the assistance of Mr. Bongani Mkhize

^b^This species appears as uvete lwaseMaskarina in Phaka et al. [30]. In this study, it was changed to uvete lwaseNile to correspond with the scientific name change of this species in South Africa [44]

### Individual species names: overlaps between folk and scientific taxonomy

Some of the isiZulu species names formulated in this study would not qualify as scientific names since they violate the principles of binomial nomenclature outlined in the ICZN Code [20]. Of the 58 isiZulu names, 33 are binomina, and the remaining names are combinations of at least three words. One of the 33 isiZulu binomina does not have its first word as the indigenous equivalent of a generic name. Not having a generic name as the first word in a species binomen violates Article 5 of the ICZN Code [20].

### Local knowledge of amphibians beyond taxonomy

Amphibians in the study area were found to have no utilitarian value as the participants confirmed that no frog species are used for culinary or cultural purposes. The participants believed that the diverse range of frog advertisement calls produced by the different Zululand species belong to insects. Common myths encountered were that grass frogs bring rain while  African clawed frogs (Pipidae) are thought to fall from the sky during torrential rain. All the participants reported that the most effective way to eradicate frogs from their homes was by throwing salt on their dorsum to make them “sweat.”

## Discussion

Scientific taxonomy aims to objectively classify all species according to their evolutionary relationships [32]. Folk taxonomy investigated in this, and other studies shows a classification system based on evolutionary groupings in nature [32], with genera being the most recognizable taxonomic level [4, 11, 33]. The folk classification investigated in this study was found to rely on frogs’ habits, habitats, or appearance when grouping species together. Ellen et al. [17] also reported that amphibian folk classification by the Nuaulu tribe of Indonesia is linked to their habits and habitats. Contrary to the current study’s findings, Nuaulu were found to use advertisement calls in their amphibian taxonomy [17]. Folk taxonomy that relies on advertisement calls is also evident in the local bird names of the Punjab province of Pakistan [1]. The finding that indigenous names collected from Zululand locals correspond to taxa higher than species (i.e., genus or family) is in line with results from some of the earliest folk taxonomy investigations which mostly focused on Oceanic and South American languages [9, 11, 12]. Bannikov [6] also obtained similar results when looking into Russian folk taxonomy of frogs. The collected folk name categories in this study were fewer than scientific taxa (see Fig. 1). This finding is similar to results from Berlin [8], which indicated that there was no exact correspondence between the number of folk and scientific taxa. When comparing the taxonomy of two Indonesian tribes, Ellen et al. [17] noted closer correspondence between amphibian folk and scientific taxa in the tribe with greater knowledge of amphibian biology. This work by Ellen et al. [17] gives an indication that the low correspondence between amphibian folk and scientific taxa may be a symptom of limited knowledge of anuran biology in the current study area.

Folk taxonomy principles used by different cultures have been found to have similarities [7]. In addition to the parallelism mentioned above, principles used in Zululand are consistent with other folk taxonomies used on various taxa by different cultures in many parts of the world including Tzeltal plant taxonomy in Mexico [11], mammalian taxonomy in the Brazilian state of Paraíba [26] and the Punjab province of Pakistan [1], mushroom taxonomy by the Maasai and Kurya of Tanzania [37], marine species taxonomy in the Ceará State of Brazil [31], and invertebrate taxonomy by ethnic Hungarians from Romania, Slovakia, and Croatia [38]. Inconsistencies also exist between Zululand frog taxonomy and other folk taxonomic systems of the world. The most notable of these is the high number of specific folk names for plants by Basotho people of Lesotho (see [25]), fish by Vaie people of Malaysia (see [19]), and Hungarian invertebrates (see [38]). To confirm whether the above consistencies and inconsistencies also apply to other indigenous South African languages requires a larger-scale investigation of amphibian folk taxonomy. The onomatopoeia, description, and imagery principles used in folk nomenclature (see [10, 24]) and outlined in this study are also evident in modern nomenclature. For instance, *Arthroleptis* applies description and imagery principles as it refers to the thin digits which are characteristic to the genus, while the generic common name Squeakers and its Afrikaans equivalent kikkers are onomatopoeic references to the genus’ advertisement calls.

The intellectualist approach to folk and scientific taxonomy, noted in this study, is an approach based on a view that human beings recognize inherent order in the natural world regardless of biota’s practical value [8]. This however does not imply that all folk taxonomy is intellectualist in its approach as other studies have reported local utilization of organisms that are subject to folk classification and nomenclature (see [31, 37, 38]). In contrast to the current study, amphibians have been previously reported to have gastronomic and traditional medicinal value in other regions of South Africa [3, 43] and many other parts of the world [2, 42].

Overlaps between folk and modern taxonomy enable the use of modern naming conventions to supplement folk taxonomy when standardizing indigenous names. This application of modern conventions to indigenous names of course needs to be done with some exceptions and within the boundaries of folk taxonomy to avoid losing the essence of indigenous names and their relevance to those who use them frequently. No classification system, modern, or indigenous, provides an infallible way of categorizing biota [8]. Thus, the abovementioned supplementation bridges gaps in folk taxonomy.

The Zulu language’s descriptive nature means that in some instances, the principles of binomial nomenclature [20] cannot be followed without affecting the meaning and appropriateness of species’ names. Thus, 25 of the 58 species names are combinations of at least 3 words while the rest are binomina. The ordering of words in the formulated isiZulu species names is another aspect of folk taxonomy that will not always conform to scientific naming rules. The isiZulu species binomen for *P. edulis*, inkunzi yexoxo, does not have a generic name as its first word as this would affect its meaning. Inkunzi used as a first word instead of the folk generic name ixoxo appropriately describes a bullish or large frog, and the resulting binomen bears a similar meaning to the species’ English name. If the folk generic name ixoxo were to be used as the first word then the resulting binomen, ixoxo yenkunzi, would merely be describing a male frog.

What seems like folklore regarding grass frogs and African clawed frogs may actually constitute an observation of amphibian behavior and attempts to explain it using available knowledge. Grass frogs may be seen moments before a rain event as the humid and moderate conditions are favorable for frog activity. If this behavior is observed repeatedly by a person without an understanding of frog biology, they may conclude that the ensuing rain was brought on by the frog seen prior to the event. African clawed frogs are aquatic species; thus, seeing them on land during or after torrential rain without knowledge of how they migrate between waterbodies may lead one to incorrectly deduce that they rained from the sky. The reported “sweating” of salted frogs is an osmotic response to the high concentration of salt the frogs’ skins are suddenly exposed to.

## Conclusions

The current study emphasizes gaps in the documentation and investigation of amphibian folk taxonomy in South Africa, while highlighting the need for standardization of indigenous frog names to increase their universality. Furthermore, this study contributes to solving two social development issues in South Africa; firstly, the need to increase public participation in biodiversity matters, and secondly, the development of indigenous languages. The research outcomes are intended not only to benefit non-scientists and but also provide a remedy to NBSAP's acknowledgement that biodiversity is not as broadly understood as it should be [34]. In line with South Africa’s Protection, Promotion, Development and Management of Indigenous Knowledge Systems Bill [35] , this research encourages the use of indigenous knowledge systems. Through increasing universality of indigenous frog names and linking their meaning to published scientific and common names, users of these indigenous names can be introduced to other names outside their home language. The guidelines used for compiling a comprehensive species list in this study are open to further improvement since they are based on the folk taxonomy of one South African language and from one area.

## Additional file

Additional file 1: Questionnaire used for folk taxonomy investigations in Zululand. Interview template used for the semi-structured questionnaire in this study. (DOCX 18 kb)

## Abbreviations

ICZN Code: International Code of Zoological Nomenclature
ICZN: International Commission on Zoological Nomenclature
NBSAP: South Africa's National Biodiversity Strategy and Action Plan
NWU: North-West University

## References

[1] Altaf M, Javid A, Umair M, Iqbal KJ, Rasheed Z, Abbasi AM (2017). Ethnomedicinal and cultural practices of mammals and birds in the vicinity of river Chenab, Punjab-Pakistan. J Ethnobiol Ethnomed.

[2] Alves RRN, Vieira WL, Santana GG, Vieira KS, Montenegro PF, Alves RRN, Rosa IL (2013). Herpetofauna used in traditional folk medicine: conservation implications. Animals in traditional folk medicine.

[3] Anthony BP, Bellinger EG (2007). Importance value of landscapes, flora and fauna to Tsonga communities in the rural areas of Limpopo province, South Africa. S Afr J Sci.

[4] Atran S, Estin P, Coley J, Medin D (1997). Generic species and basic levels: essence and appearance in folk biology. J Ethnobiol.

[5] Auerbach R (1986). First steps in Setswana herpetology. Botswana Notes and Records.

[6] Bannikov AG (1971). Amphibians and reptiles of the USSR.

[7] Berlin B (1973). Folk systematics in relation to biological classification and nomenclature. Annu Rev Eco Evol Syst.

[8] Berlin B (1991). The chicken and the egg-head revisited: further evidence for the intellectualist bases of ethnobiological classification. In: man and a half: essays in pacific anthropology and ethnobiology in honour of Ralph Bulmer. J Polyn Soc Memoirs (Dd.).

[9] Berlin B (1992). Ethnobiological classification: principals of categorization of plants and animals in traditional societies.

[10] Berlin B. The first congress of ethnozoological nomenclature. J Royal Anthropol Inst. 2006;12:S23–44.

[11] Berlin B, Breedlove DE, Raven P (1973). General principles of classification and nomenclature in folk biology. Am Anthropol.

[12] Brown CH (1979). Folk zoological life-forms: their universality and growth. Am Anthropol.

[13] da Silva MJ, Barbosa Filho ML, Alves RRN, Albuquerque UP (2018). Ethnotaxomy as a methodological tool for studies of the ichthyofauna and its conservation implications: a review. Ethnozoology: animals in our lives.

[14] Dougherty J (1979). Learning names for plants and plants for names. J Linguist Anthropol.

[15] Du Preez LH, Carruthers VC (2009). A complete guide to the frogs of Southern Africa.

[16] Du Preez LH, Carruthers VC (2017). Frogs of Southern Africa.

[17] Ellen RF, Stimson A, Menzies J (1976). Structure and inconsistency in Nuaulu categories for amphibians. J Agric Tradit Bot Appl.

[18] Harrison KD (2007). When languages die: the extinction of the world’s languages and the erosion of human knowledge.

[19] Hidayati S, Ghani BA, Giridharan B, Hassan MZ, Franco FM (2018). Using ethnotaxonomy to assess traditional knowledge and language vitality: a case study with the Vaie people of Sarawak, Malaysia. Ethnobiology Letters.

[20] ICZN (1999). (International Commission on Zoological Nomenclature). International Code of Zoological Nomenclature.

[21] Jacobsen N (1978). Colloquial names for southern African reptiles and amphibians. Afr J Herpetol.

[22] López A, Atran S, Coley JD, Medin DL, Smith EE (1997). The tree of life: universal and cultural features of folk biological taxonomies and inductions. J Cogn Psychol.

[23] Minter LR, Burger M, Harrison JA, Braack HH, Bishop PJ, Kloepfer D (2004). Atlas and red data book of the frogs of South Africa, Lesotho and Swaziland. SI/MAB series #9.

[24] Möller LA (2017). Of the same breath: indigenous animal and place names.

[25] Moteetee A, Van Wyk BE (2006). Sesotho names for exotic and indigenous edible plants in southern Africa. Bothalia..

[26] Mourão JS, Araujo HF, Almeida FS (2006). Ethnotaxonomy of mastofauna as practised by hunters of the municipality of Paulista, state of Paraíba-Brazil. J Ethnobiol Ethnomed.

[27] O’Brien CM (2010). Do they really “know nothing”? An inquiry into ethnobotanical knowledge of learners in Arizona USA. Ethnobot Res Appl.

[28] Passmore NI, Carruthers VC (1978). Further comment on English common names for south African frogs. Afr J Herpetol.

[29] Passmore NI, Carruthers VC (1979). South African frogs.

[30] Phaka FM, Netherlands EC, Kruger DJD, Du Preez LH (2017). A bilingual field guide to the frogs of Zululand. Suricata 3.

[31] Pinto MF, da Silva MJ, Alves RR (2013). Ethnotaxonomical considerations and usage of ichthyofauna in a fishing community in Ceará State, Northeast Brazil. J Ethnobiol Ethnomed.

[32] Ross NJ, Quave CL (2014). “What’s that called?” folk taxonomy and connecting students to the human-nature interface. Innovative strategies for teaching in the plant sciences.

[33] Savo V, Bisceglie S, Caneva G, Kumbaric A, Mcclatchey WC, Reedy D (2011). “Modern Linnaeus”: a class exercise on plant nomenclature and taxonomy in comparison with a previous experiment. Ethnobot Res Appl.

[34] South Africa's Department of Environmental Affairs (DEA). 2015. South Africa’s National Biodiversity Strategy and Action Plan 2015 - 2025. https://www.environment.gov.za/sites/default/files/docs/publications/SAsnationalbiodiversity_strategyandactionplan2015_2025.pdf. Accessed 21 Apr 2018.

[35] South Africa. 2016. Protection, promotion, development and management of Indigenous Knowledge Systems Bill (B6–2016).

[36] Tarrant J (2015). My first book of southern African frogs.

[37] Tibuhwa DD (2012). Folk taxonomy and use of mushrooms in communities around Ngorongoro and Serengeti National Park, Tanzania. J Ethnobiol Ethnomed.

[38] Ulicsni V, Svanberg I, Molnár Z (2016). Folk knowledge of invertebrates in Central Europe-folk taxonomy, nomenclature, medicinal and other uses, folklore, and nature conservation. J Ethnobiol Ethnomed.

[39] Van Dijk DE (1978). Comments on English names for southern African Anura. Afr J Herpetol.

[40] Van Dijk DE (1978). English names for Southern African Anurans. Afr J Herpetol.

[41] Vesey-Fitzgerald D (1960). Vernacular names. J Herpetol Assoc Rhodesia.

[42] Warkentin IG, Bickford D, Sodhi NS, Bradshaw CJ (2009). Eating frogs to extinction. Conserv Biol.

[43] Williams VL, Whiting MJ (2016). A picture of health? Animal use and the Faraday traditional medicine market, South Africa. J Ethnopharmacol.

[44] Zimkus BM, Lawson LP, Barej MF, Barratt CD, Channing A, Dash KM (2017). Leapfrogging into new territory: how Mascarene ridged frogs diversified across Africa and Madagascar to maintain their ecological niche. Mol Phylogenetics Evol.

