# Supplementary material for: Folk taxonomy and indigenous names for frogs in Zululand, South Africa
Source: J Ethnobiol Ethnomed. 2019 Mar 26;15:17. doi: 10.1186/s13002-019-0294-3 (PMC6434812; doi:10.1186/s13002-019-0294-3)
Supplement: Supplementary file 1 — Questionnaire used for folk taxonomy investigations in Zululand. Interview template used for the semi-structured questionnaire in this study. (DOCX 18 kb) [file 13002_2019_294_MOESM1_ESM.docx]

| Additional file 1: Questionnaire used for folk taxonomy investigations in Zululand | |
| --- | --- |
|  | |
| 1. Do you know any isiZulu names for the pictured frogs? | |
| Yes | No |
|  |  |
| 2. If you replied ‘yes’ to the previous question, provide isiZulu names for the pictured frogs. | |
|  |  |
|  |  |
| 3. What is the reasoning or meaning behind the isiZulu name(s) you provided? | |
|  |  |
|  |  |
| 4. Do Zululand locals use frogs for personal or cultural purposes? | |
| Yes | No |
|  |  |
| 5. If you replied ‘yes’ to the previous question, point out the picture of the frog and describe what it is used for. | |
|  |  |
|  |  |
